# Understanding adaptation to digital transformation: evidence from Indian advertising industry

**DOI:** 10.3389/fsoc.2026.1953897

**Published:** 2026-10-28

**Authors:** Pranav S. Joshi, Nandini Lakshmikantha

**Affiliations:** 1 Manipal Institute of Communication, Manipal Academy of Higher Education, Manipal, India; 2 Manipal Institute of Technology, Manipal Academy of Higher Education, Manipal, India

**Keywords:** adaptation, advertising professionals, digital transformation, organizational tensions, work transformation

## Abstract

**Introduction:**

Digital Transformation (DT) is changing the advertising industry by influencing how professionals design their campaign strategies, cooperate with clients, collaborate with teams, and develop new skills in response to technological and market demands. While the existing literature has primarily focused on consumers and advertising platforms, it has given limited attention to the lived experience of advertising professionals as they adapt to and navigate DT. This study fills this gap by exploring how Indian advertising practitioners comprehend and interpret organizational adaptation to DT.

**Method:**

The present research employed a qualitative approach, using in-depth semi-structured interviews with 40 advertising professionals selected through purposive sampling across different roles. Data were analyzed using inductive thematic analysis conducted through ATLAS.ti to develop themes explaining how advertising practitioners experience DT.

**Result:**

The analysis revealed four themes: disruption forces, tensions within the industry, adaptation, and transformation of advertising work. Together, they conceptualize DT as a dynamic and interconnected process rather than a series of isolated changes. The findings revealed tensions centered on technological complexity, extensive consumer data, budget limitations, changing consumer behavior, threats from emerging digital agencies, and a lack of digital talent. In response, professionals are adopting digital tools, embracing flexible work practices, and reorganizing their agency structure to stay agile and competitive. However, as working practices continue to evolve in a digital environment, they generate new tensions that require further adaptation.

**Discussion:**

A Recursive Tension—Adaptation Framework, a conceptual model, is proposed based on an analysis of Indian Advertising Professionals’ experiences, explaining how professionals within advertising organizations continuously adapt to an evolving digital work environment and offering implications for managing organizational change and workforce development.

## 1 Introduction

In today’s world, marked by the rapid proliferation of disruptive technologies, ongoing Digital Transformation (DT) has become a defining force in organizational operations and strategy (Ioniță et al., 2025). Companies of all sizes and industries are now pursuing DT to improve operations and create value through innovation. Startups, SMEs, and players from traditional industries are also considering reshaping their capabilities, shifting priorities, and advancing through digital approaches to compete in the digital economy (Facin et al., 2022). As a result, the growing prominence of DT in business environments has attracted increasing scholarly attention in the business literature (Ali et al., 2023; Cioppi et al., 2023).

DT is the process of incorporating digital tools across all aspects of a company’s operations and tactics, creating new value for customers while simultaneously enhancing internal efficiency (Sundaram et al., 2020). From an advertising viewpoint, DT goes beyond replacing traditional methods to using a range of methods and technologies. It supports integrating digital technologies into advertising strategies to improve effectiveness and the customer experience (Pratama, 2025). It has introduced data-driven, evidence-based techniques that focus primarily on consumer behavior (Thota et al., 2023). A variety of formats, including Social Media Marketing (SMM), Search Engine Optimization (SEO), content marketing, email marketing, pay-per-click, affiliate marketing, targeted ads, and changing user characteristics, are crucial for businesses to operate successfully within an increasingly digital economy (Zhou, 2023; Nandi and Subrahmanyan, 2024).

The rapid growth of digital technologies and the resulting shifts in the competitive and consumer landscape have forced companies to adopt relevant DT (Ceccotti et al., 2025). This trend is noticeable in India, a country recognized as one of the largest economies in the world, which is experiencing DT across multiple sectors, from manufacturing and service industries to small and medium-sized enterprises (SMEs) (Gehlot et al., 2024). A recent report from the Federation of Indian Chambers of Commerce and Industry (FICCI) highlights a significant transformation in India's advertising service industry driven by digital technology. While advertising has increased to INR 1.38 trillion, with digital media growing at 11%, digital is forecast to account for 61% by 2027. This shift, driven by advances in Artificial Intelligence (AI), performance marketing, social media apps, and digital devices, has enabled businesses to grow more quickly, raise their advertising investment, and embrace new digital media, prompting traditional organizations to adapt to a new business model (FICCI and EY India, 2025). As digital technologies continue to influence the advertising ecosystem (Maurya, 2025), both traditional and digital agencies must redesign their strategies to remain competitive (Gowda and Raja, 2026). Considering these developments, the current study explores DT in the Indian advertising sector from a professional perspective.

Existing research has demonstrated the effects of DT on advertising agencies across various dimensions, including changes and challenges in organizational structure, stakeholder relationships, industry practices, job roles, required skill sets, emerging digital technologies, and their integration into advertising work, with a focus on data-driven decision-making and cross-functional collaboration. (Childers et al., 2018; Windels and Stuhlfaut, 2018; Hughes and Vafeas, 2019; Patwardhan et al., 2019; Pascucci et al., 2023; Mokoena et al., 2023; Ceccotti et al., 2025). Previous research shows that DT extends beyond adopting digital technologies and influences how advertising agencies and professionals operate. Despite these developments, existing knowledge remains fragmented and offers limited understanding of how these dimensions interact in organizational adaptation to DT. Furthermore, while understanding DT, extensive knowledgebase have been developed from the perspective of consumer (Alghizzawi, 2019; Rasool et al., 2020; Saura et al., 2020; Roy et al., 2023; Chan, 2024; Jain et al., 2024; Ambika et al., 2025; Tan et al., 2025) and advertising platforms (Kumar Sharma et al., 2020; Gao et al., 2021; Krishen et al., 2021; Ghorbani et al., 2022; Shaheen, 2025) whereas limited attention has been given to the lived experiences of advertising professionals who come across these changes in their everyday work practices. Various scholars have also highlighted this gap, emphasizing the need to understand DT through industry practitioners' experiences (Moeran, 2015; Cioppi et al., 2023; Pascucci et al., 2023; Plekhanov et al., 2023; Ceccotti et al., 2025).

Addressing this gap has become vital, as DT is not simply the implementation of technology. It is shaped by the ability of practitioners to comprehend evolving technologies, understand and adapt to them while incorporating in routine existing work practices. Examining this gap will provide a more holistic understanding of DT as an organizational process rather than a simple transition. Against this background, the following question is formulated.

*RQ*: How do advertising professionals comprehend and interpret organizational adaptation to digital transformation within the Indian advertising industry?

The present study makes three crucial contributions to the existing knowledge base. First, it provides professional-based empirical evidence from the Indian advertising industry, extending DT knowledge within a rapidly evolving digital landscape. It then contributes by bringing together four dimensions—digital disruption forces, professional and organizational tensions, adaptation mechanisms, and transformation of advertising work to elaborate on how they interact with each other instead of as isolated changes. Lastly, the study identifies a recursive tension—adaptation framework that conceptualizes DT as an ongoing process in which digital disruption generates tensions, organizations respond through adaptation, and transformed work generates further tensions in the process. In this way, the present study suggests a process-oriented understanding of how professionals continuously navigate DT.

To address research question, the remainder of the manuscript is structured as follows: the literature review examines the influence of digital technologies on the advertising industry. This is followed by the methodology, which outlines the research design. The results section explains each theme and proposed framework that emerged from thematic analysis, followed by a discussion, implications, limitations, future scope, and conclusion.

## 2 Literature review

### 2.1 Digital transformation in advertising

Advertising has experienced a remarkable transformation due to several technological innovations such as Internet of Things (IoT), AI, blockchain, big data analytics, 4G/5G internet speeds, social media, mobile technologies, automation, and other interactive digital technologies (Faruk et al., 2021; Pellegrino, 2024), transforming simple banner ads into diverse, data-driven formats and a performance-based business model (Zhou, 2023). Subsequently, advertising agencies which is central organization within advertising industry, specialized in creating and executing advertising campaigns using various media (Agarwal et al., 2023), are adopting new trends such as SEO, paid search, SMM, influencer marketing, IoT, cloud computing, Augmented Reality (AR), and data analytics, thereby strengthening their digital capabilities and optimizing campaign performance (Caliskan et al., 2021; Yadav and Reddy, 2023; Kumar et al., 2024). This has attracted many researchers to understand their applications, use for consumer insights, advertising effectiveness, impact on creativity, brand engagement analysis, advertising performance, and consumer purchase intentions (Kumar et al., 2024).

### 2.2 Digital transformation and advertising agencies

Incorporating digital media into the marketing function has compelled agencies to adopt new technologies, necessitating a strategic shift in the business framework and aligning the digital approach with overall business strategy. (Rathore, 2019). This has reshaped value propositions, value creation, customer relationships, service portfolios, organizational structures, and emerging professional roles (Ceccotti et al., 2025). The COVID-19 pandemic further accelerated DT, compelling organizations to redesign their strategies and operating models through the extensive implementation of digital technologies and necessitating an analysis of DT (Cioppi et al., 2023). Similar organizational changes have been observed across different national contexts. For example, Spanish agencies saw changes in gender dynamics as DT reduced traditional gender disparities (Papí-Gálvez et al., 2020), while Italian industries saw the emergence of multidisciplinary marketing roles, shifting from simple communication functions to key strategic functions with advanced intelligence and analytical tasks (Pascucci et al., 2023).

These developments have increased the significance of data-driven managerial decision-making. Marketing managers must leverage big data, AI and Machine Learning (ML) to predict future consumer behavior, replace intuition-based decisions with evidence-based strategies, and enhance customer experience through real-time analytics (Figueiredo et al., 2021; Prihatiningsih et al., 2024). Similarly, a study of 100 advertising professionals at a traditional creative agency found that advanced tools significantly affected their campaigns, highlighting their need to learn new programming skills. At the same time, their adoption also introduced a range of psychological, social, and organizational constraints for practitioners, including job replacement anxiety, steep learning requirements, privacy and ethical issues, team collaboration, managerial support, and training and upskilling (Iyer and Bright, 2024).

DT has also reconfigured the agency-client relationship, with clients increasingly internalizing key services such as website development, data analysis, and social media through digital capabilities while outsourcing creativity to external agencies. This shift has compelled agencies to clearly differentiate their value creation and value propositions (Hughes and Vafeas, 2019; Mokoena et al., 2023). From a leadership perspective, US agencies have experienced pressures externally ranging from client expectations to shorter agency-client relationships while internally, agencies struggle with digitally skilled talent, traditional organizational structures and human resource challenges. However, successful digital agencies responded by strategically repositioning at the intersection of technology and marketing, cross-platform training, and hiring fresh digital talent (Patwardhan et al., 2019).

The creative and media landscape has undergone a similar transition. New interactive, dynamic media have shifted business practices from linear workflows to collaborative, technology-driven processes, resulting in new specialist roles, integration of digital knowledge throughout the creative process, organizational restructuring, expanded service portfolios, and increasing demand for technical expertise across “management, strategy, creative, and production” (Windels and Stuhlfaut, 2018; Lynch, 2019; Stuhlfaut and Windels, 2019). Similar trends were also seen in South African agencies. Digital disruption was perceived as a challenge as it threatened established models of value creation. It emphasized the need to integrate creativity, technology, data, and omnichannel strategies through cohesive, digitally enabled processes (Mokoena et al., 2023). Likewise, senior leaders in traditional full-service agencies noted that departments are evolving to meet digital demands, creating both opportunities and obstacles. On one hand, advancements enable precise targeting, real-time analytics, and more accurate consumer understanding through automation; on the other, organizational restructuring has led to fragmentation and reduced creative autonomy as new, specialized, and overlapping job roles become common. Additionally, inflexible client-side processes prevent dynamic and responsive budgeting practices (Childers et al., 2018).

In summary, DT extends beyond technology adoption to reshape the organizational and professional landscape of advertising agencies, and further research is needed to understand how these changes play out in specific national contexts, particularly in India.

### 2.3 Indian advertising agencies

In Indian context, earlier research largely focused on peculiar organizational functions and management practices. It included challenges associated with digitalization, new media, unorganized market landscape, profit margin (Joshi et al., 2011), analytical based forward-looking approach instead of intuition-based decisions (Xavier et al., 2011), account planning function (Patwardhan et al., 2011), practitioners’ perceptions and reflections on consumer culture in campaigns (Swaminathan and Bansal, 2014), development of strategic capabilities due to green marketing innovations (Kumar, 2015), practical challenges for practitioners associated with rural marketing such as brand consciousness, limited infrastructure, cultural variety, fluctuating demand, and managing the sales team (Parida and Sahney, 2018), creative perspective for ad agency leadership (Gaur, 2016) and quality management practices for increasing ad agency efficiency, creativity and innovation (Aarzoo and Lal, 2025). While these studies offer important professional insights, they largely focus on specific managerial concerns rather than addressing a broader DT perspective.

The impact of DT goes beyond advertising firms to the wider marketing landscape. Senior marketing professionals in SMEs have shared that the lack of IT infrastructure and slow technology adoption are major hurdles to restructuring their marketing strategies (Muthiah, 2006). Similarly, Kumar Sharma et al. (2020) found that digital marketing agencies working with SMEs encountered challenges arising from limited knowledge of digital practices, unrealistic expectations, dissatisfaction from comparison with large firms, budget restrictions, and operational delays. Despite these obstacles, SMEs were seen as an untapped market because of their simpler structures, expansion ambitions, and opportunities to build relationships through education. Similarly, Shukla (2018) observed the underutilization of marketing divisions and their employees in the IT industry, suggesting that marketing professionals could play a strategic role with adequate human and technological resources. Furthermore, big data analytics (BDA) and social media analytics (SMA) have been considered as enablers for data-driven marketing strategies that can enhance customer engagement using their real-time data, enhancing organizational performance across industries like FMCG, retail, and IT (Garg et al., 2020; Mukhopadhyay et al., 2024). Similar developments are becoming increasingly evident in advertising agencies.

In recent years, the growth of social media, influencers, and AI in India has changed the advertising industry landscape. This shift is evident in the move from mass to digital media, advertising methods, creativity, job responsibilities, and analytical and promotional competences (Pharswan et al., 2020; Kumar et al., 2024; Rathod et al., 2022). Although these technologies provide employment prospects for young talent (Malik et al., 2022) and enable effective campaign creation and optimization, tools such as AI also alarms job security in repetitive tasks and human creativity (Surana, 2025). Subsequently, adapting to these tools has highlighted the critical need for workforce capability development, necessitating continuous upskilling, balancing new technology with human talent, training and educational programs, and updating academic curricula (Saini et al., 2026). While tools like cloud computing and analytics have enhanced operational efficiency, they have also created challenges such as developing new technical capabilities, work-life balance, strengthening emotional intelligence, and fostering creative customer relationships, which organizations need to address (Ali et al., 2023).

Overall, existing literature shows that DT has influenced advertising companies across multiple dimensions, such as work practices, technology, organizational structure, agency-client relationships, capabilities, and emerging job roles. Together, these studies indicate that technological developments have led to broader changes in the advertising industry. This provides a foundation for examining organizational adaptation to DT from advertising professionals' perspective.

## 3 Methods

### 3.1 Research design

The study adopted qualitative research design, as shown in Figure 1, to obtain a deep, contextual understanding of participants’ viewpoints, experiences, perceptions, and meanings (Lim, 2025). To address the research question, the study employed in-depth semi-structured interviews conducted by the corresponding author to explore professionals’ perspectives, as this method enables participants to convey the experiences in their own words (Golam Azam, 2022). The authors followed inductive thematic analysis in the present study. This approach is ideal when researchers prepare, read, and code raw data to identify main themes through recurring patterns, without requiring predetermined theories or hypotheses, unlike the deductive approach, which examines existing theories. The inductive thematic approach allowed theory to emerge from the data (Thomas, 2006).

**Figure 1:**
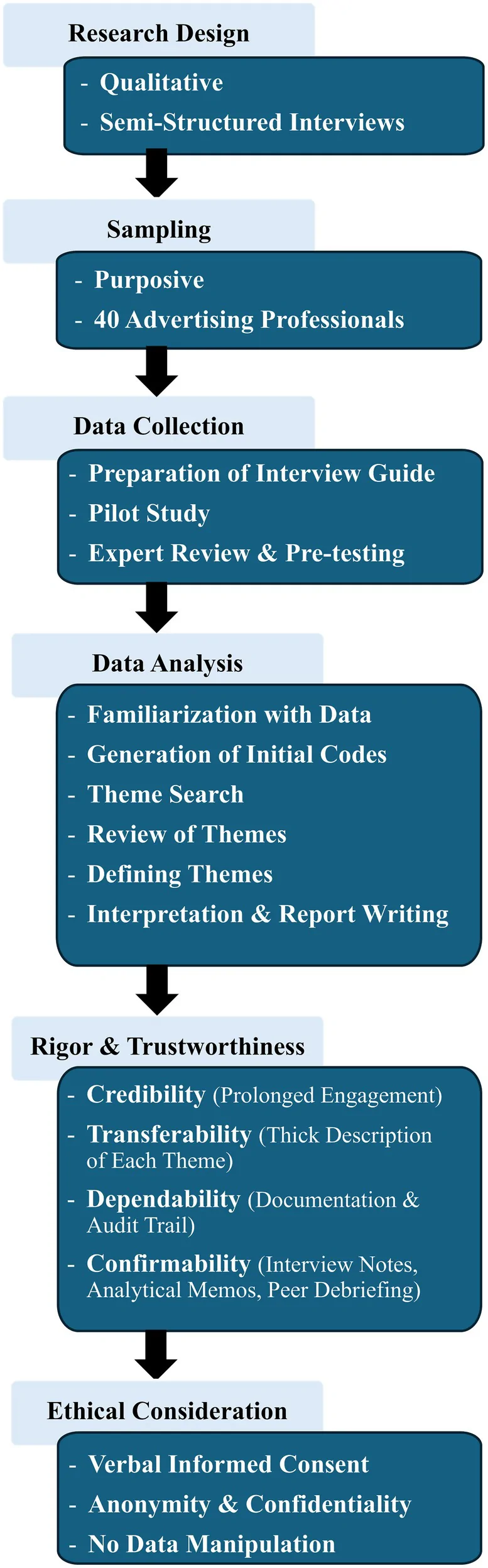
Overview of research process adopted in the study.

### 3.2 Sampling

According to Braun and Clarke (2021), thematic analysis does not have a fixed sample size; rather, sample adequacy depends on research objectives, participant diversity, and the richness and depth of the data. Interviews continued until no new themes or insights emerged (Hennink and Kaiser, 2022), resulting in a final sample size of 40 advertising professionals. After every interview, notes were recorded in a saturation-monitoring log whenever patterns recurred, to ensure data saturation. Additionally, ATLAS.ti software was used to track code recurrence in each interview (Ahmed, 2025).

Purposive sampling was used to select knowledgeable and experienced participants, yielding valuable, experiential, and diverse insights. Participants were selected from both established and newly formed digital agencies across various hierarchical levels, allowing the study to capture valuable and diverse insights. No restrictions were placed on age, gender, geographic setting, or job roles (Ahmad and Wilkins, 2025). Participants’ profiles are provided in Table 1.

**Table 1:** Participant’s profile.

No	Gender	Age	Designation
1	Male	33	Creative account director
2	Male	46	General manager—digital CRM
3	Male	41	Business head—digital
4	Female	34	Associate account director
5	Male	28	Digital marketing strategist
6	Male	42	Senior business director
7	Female	49	Managing partner
8	Male	24	Copywriter
9	Female	60	Senior vice president
10	Male	52	Executive director
11	Male	48	President
12	Male	35	Director—strategy and content
13	Male	55	CEO
14	Male	55	Executive director
15	Female	32	New business development lead
16	Male	33	Associate media director
17	Female	37	Design head
18	Male	32	Group account manager
19	Male	27	Media planner
20	Male	39	Co-founder & MD
21	Male	32	Co-founder & CEO
22	Male	41	Head of digital
23	Male	23	Assistant director
24	Male	30	Senior account director
25	Male	45	Senior vice president & country head
26	Male	28	Senior associate—PR & influence
27	Female	20	Copywriter
28	Male	31	Brand executive
29	Female	23	Account executive—digital reputation
30	Male	35	Brand service director
31	Male	26	Digital marketing head
32	Male	47	Founder
33	Male	31	Account controller
34	Female	37	Business director
35	Male	30	Media planner
36	Female	33	Media planner
37	Female	22	Account executive—digital strategy & servicing
38	Male	32	Digital marketing executive
39	Male	40	Vice president
40	Male	60	National creative director

Source: author developed.

### 3.3 Data collection

To gather data, authors developed an interview guide to frame the topic and guide participants’ thinking, yielding richer data (Naz et al., 2022). After identifying the prerequisites for semi-structured interviews based on the theoretical background and research objectives, the researchers prepared a comprehensive interview guide in four sections (Roberts, 2020). The guide underwent academic expert review, and the researchers pre-tested the questions using the modified Question Appraisal System-99 (QAS-99) (Willis and Lessler, 1999). It helped to achieve the questionnaire's trustworthiness and reliability (Bhalla et al., 2023). A pilot study comprising 5 practitioners was conducted to assess the interview guide and support the research design. Based on these interviews, minor refinements were made to improve question wording and sequencing of selected questions. The interview guide was methodically structured into four sections to ensure a logical progression of questions and support a conversational flow. No major changes were made to the core questions, topics covered, or research focus after the pilot study and all interviews were retained for final analysis. (Malmqvist et al., 2019). The accompanying dataset includes the interview guide (Joshi, 2026). Each interview was conducted in English, and lasted between 40 min to1 hour 10 min, averaging about 50 min. After basic demographic questions, the interview section began with participants narrating their initial experiences with advertising technology and its status. Later, the corresponding author asked key questions to clarify the research objectives. This allowed to gain a real-world perspective of how digital technology is influencing the Indian Advertising Industry.

### 3.4 Data analysis

The corresponding author analyzed the collected data using inductive thematic analysis (Braun and Clarke, 2006; Proudfoot, 2023), which helped identify four main themes. Thematic analysis consists of the following six steps. First, data were transcribed from recordings into text using Microsoft (MS) Word to become familiar with the data, then imported into ATLAS.ti. Authors used licensed student versions of the software as it is one of the widely used tools that facilitates efficient management and organizations of interview transcript, coding, categories, quotations and memos. Considering substantiable interview data of 40 participants, this tool was obvious choice which helped with systematic coding. Features like code manager helped in delivering more robust and coherent results (Adelowotan, 2021). Authors, with the support of software developed 1,209 initial or independent codes. These codes act as label to the quotation for which it is applied. Code manager feature helped in managing and organizing these codes which displayed quotation and codes. Code density for each quotation was noted down to identify number of distinct or conceptually similar codes. Quotations with maximum densities were selected to examine for further stages of analysis. In the third step, the codes were iteratively reviewed to identify recurring patterns and potential thematic groupings. Relevant quotations for which code was applied were read and reviewed to find the pattern using the co-occurrence tool. This paved the way for developing initial themes. Next, the developed themes were reviewed against the study's research objective, and a further round of coding was conducted, combining software-assisted and manual analyses of the transcripts. All the initial codes were then grouped and organized into 27 categories to avoid duplication. This helped identify patterns, from which four themes and their subthemes were developed. Each theme and subtheme was defined based on its underlying essence and assigned a concise title. In the final step in thematic analysis authors produced a report with detailed explanations for each theme in the form of proposed framework in the next section (Braun and Clarke, 2006; Zairul, 2025). Separate interview transcripts, initial codes, categorized codes are provided in the accompanying database (Joshi, 2026).

### 3.5 Rigor and trustworthiness

Because the present study adopted a qualitative approach, rigor and trustworthiness were established to ensure systematic, methodical conduct and assessed using four criteria, as shown in Table 2.

**Table 2:** Rigor and trustworthiness.

Criteria	Implementation
Credibility	Prolonged engagement with both participants and data (Ahmed, 2024; Lim, 2025).
Transferability	Thick description of each theme and with a carefully selected sample (Ahmed, 2024; Lim, 2025).
Dependability	Comprehensive methodological documentation and audit trail through records of raw data, interview recordings, codes and relevant documents (Lim, 2025).
Confirmability	Through interview notes, analytical memos, and peer debriefing (Gunbayi, 2024; Lim, 2025).

Source: author developed.

### 3.6 Ethical considerations

The study upheld ethical principles throughout using various strategies, as shown in Table 3.

**Table 3:** Ethical consideration.

Criteria	Implementation
Informed consent	Verbal consent was acquired before the commencement of each interview. Written consent was not feasible due to participants' geographic dispersion. All participants were informed about the purpose, nature, and scope of the study with the assurance of their right to withdraw from the study at any stage without consequences (Pietilä et al., 2020; Nii Laryeafio and Ogbewe, 2023).
Confidentiality and anonymity	Participants’ demographic information was kept confidential and anonymous throughout the study (Pietilä et al., 2020), and they were assigned identification codes from R1-R40
Consent for recording	Interviews were audio-recorded only after obtaining prior verbal approval from participants (Nii Laryeafio and Ogbewe, 2023).
Accuracy and integrity of data	No form of alteration or manipulation was undertaken during any stage of research (Kang and Hwang, 2021).
Authentic representation of responses	Participants’ intended meaning was preserved during data analysis. Direct quotes were included wherever required (Kang and Hwang, 2021).
Research relationship	No prior personal or professional relationship existed between researchers and participants, with interactions strictly within the scope of the research.

Source: author developed.

## 4 Results

This study aims to explain how professionals within advertising agencies understand and interpret organizational adaptation to DT. Thematic analysis revealed four higher-order themes: Digital Disruption Forces, Professional and Organizational Tensions, Adaptation Mechanisms, and Transformation of Advertising Work. These themes are interconnected and form the empirical basis of the proposed Recursive Tension–Adaptation Framework, shown in Figure 2, which explains how disruption generates tensions that prompt adaptive responses and resulting transformation work, leading to new conditions that require further adaptation. This reflects the recursive nature of the process and is elaborated in the following sections. To demonstrate more clarity on theme development, an illustrative example for the theme—Adaptation mechanism is provided in Table 4 in the Supplementary material.

**Figure 2:**
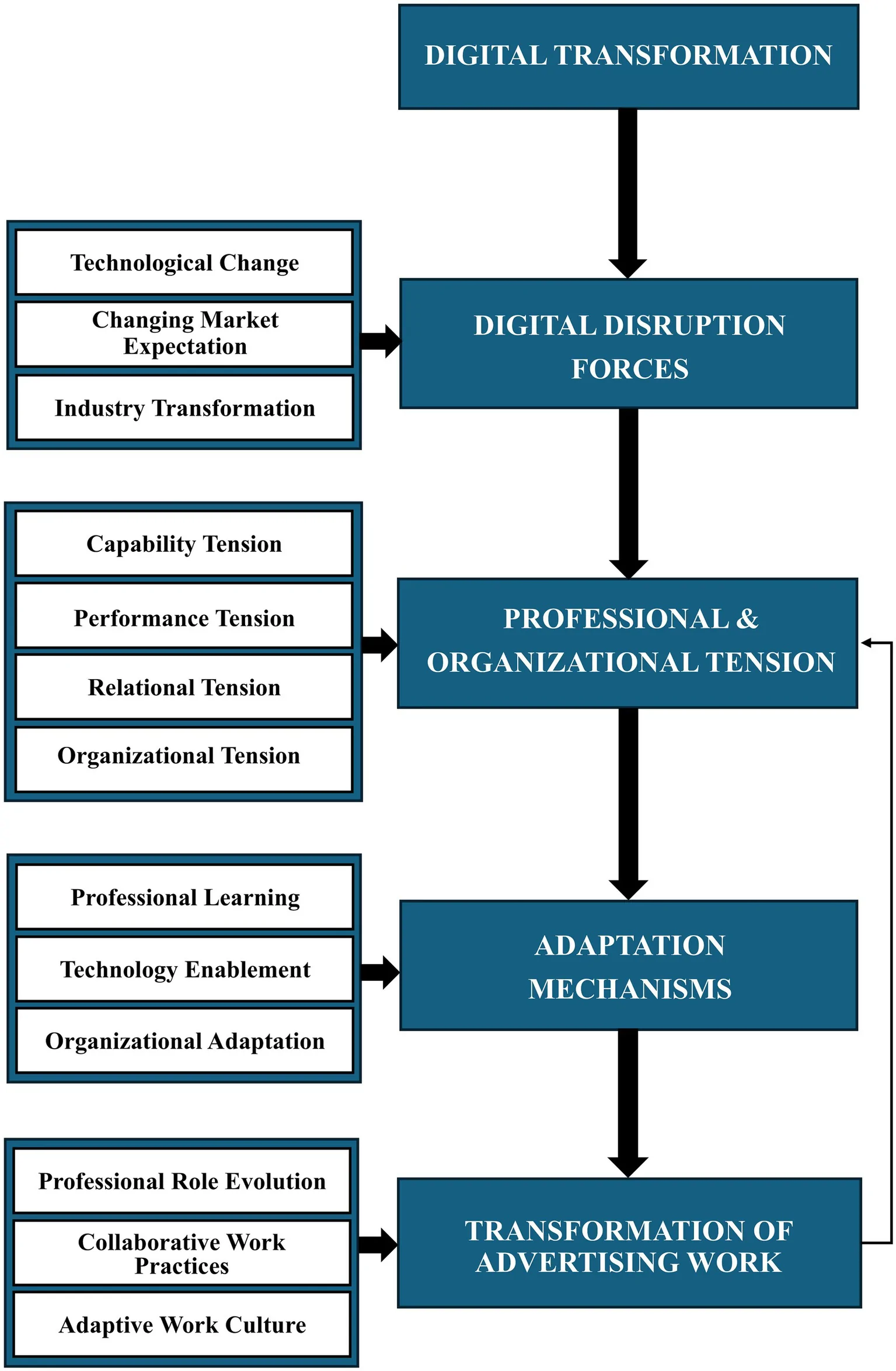
Recursive tension—adaptation framework.

### 4.1 Digital disruption forces

Digital disruption forces are external influences that change an agency's operations and value creation. These forces underpin organizational change, creating pressure that can be seen as tension in ad agencies. Three key forces that arose from the analysis are:

#### 4.1.1 Technological change

The rapid growth of digital technologies like AI, social media analytics, automation, and performance tools has transformed how agencies work. Respondents emphasized that agencies are no longer working with inadequate information but with large, constantly changing consumer data. This intensification creates more opportunities for precise targeting and measurement, yet it adds layers of technological complexity to the advertising landscape, requiring continuous adaptation.

#### 4.1.2 Changing market expectations

DT has expanded the range of media channels, platforms, formats, and methods of consumer engagement. Agencies now operate within a complex network of social media, mobile applications, search engines, OTT platforms, and e-commerce sites. This forces agencies to change how they work. Today’s Consumers and clients expect not only faster personalized work but also measurable, tailored, platform-specific work. As a result, the market landscape has become more dynamic, fast-moving, and difficult to manage.

#### 4.1.3 Industry transformation

New competition emerges as technology advances. Newly specialized, digitally focused agencies have disrupted the industry landscape, providing niche services like SEO, SMM, Influencer marketing, and data analytics. At the same time, agency-client relations have taken a new form as clients develop in-house capabilities for various advertising methods such as analytics and social media operations. This shift has reduced entry barriers while creating new business dynamics, compelling traditional agencies to distinguish themselves in a crowded marketplace. Respondents also identified broader institutional changes, including changing government regulations, digital infrastructure development, and the COVID-19 pandemic. At the center of DT

Together, these forces reshape the way advertising professionals manage their work. They contribute to a dynamic operating environment, generating internal professional and organizational tensions through which DT is experienced and managed.

### 4.2 Professional and organizational tension

DT in the Indian advertising industry has brought substantial technological advancement, but it has also generated professional and organizational tensions. The term tensions refers to conflicting forces and inconsistencies within the organization that must be managed continuously (Dieste et al., 2022). These tensions reflect deeper structural and strategic misalignment within agencies.

#### 4.2.1 Capability tension

It results from the increasing gap between demand for advanced technical knowledge and the limited pool of relevant skills in the workforce. As advertising practices depend more on data analytics, AI, and other digital tools, the range of required proficiencies also increases. This places pressure on the workforce to update their skill set. As highlighted by a respondent

“Advertising agencies now have a huge amount of consumer data, and companies do not know how to handle it.” (R2)

This suggests that advertising agencies struggle with translating complex consumer data into meaningful insights. Rapid technological change also supports this, as it makes previous knowledge obsolete and deepens this tension. One respondent noted

“The technology or advertising method learned 3–4 years back may not be used today.” (R7)

This underlines that DT not only improves analytical opportunities but also increases the pressure on agencies to develop new competencies, forcing agencies to remain flexible and adapt to changing technology. Furthermore, continuous learning has emerged as a key factor driving this tension. Even after hiring skilled professionals, employees must adapt to new digital tools. They need to identify effective strategies by learning what works and what doesn’t with these digital tools. As observed by a respondent,

“Technology innovation has changed the advertising game, and it will keep on changing in the future as well.” (R1)

This makes capability development an ongoing and uncertain process. Education is another critical factor contributing to capability tension, as a gap exists between practical training on current market trends and curricula in educational institutes, leading to incompetent practitioners entering the industry. Hence, agencies need to invest in resources for training and upskilling of professionals.

#### 4.2.2 Performance tension

Performance tension arises from performance expectations, budget allocation, and financial uncertainty. Agencies are now expected to deliver and justify measurable outcomes through data-driven metrics. However, this expectation is complicated as

“Clients lack a clear understanding of required investment” (R8)

for these digital campaigns and the ways to evaluate their returns. Respondents also pointed to budget allocation as a persistent issue, especially for smaller clients trying to replicate larger firms' strategies without adequate financial capacity. Accurately justifying return on investment (ROI) has become a tedious job because of complex modern digital tools such as programmatic buying, keyword auctions, and analytics. This further creates tension between accountability and uncertainty, as agencies must justify their expenditure while operating with restricted budgets. As a respondent said,

“Budget cutting was not specific to digital media, but it was overall” (R9)

making it challenging for agencies to balance investment across traditional and digital domains. Increased client awareness of digital performance and growing expectations for real-time performance and measurable outcomes have changed the traditional approach altogether. One respondent observed,

“Clients now ask questions such as why allocate money on the platform and why not on other platforms” (R17)

indicating the client’s involvement in decision-making processes. In addition, participants also observed more demand for digital presence, deeper analytical insights, and faster execution. In many cases, clients impose predefined Key Performance Indicators (KPIs), which means

“The agency must go back, work those KPIs, and find the best possible solution.” (R17)

This indicates the need to align media planning and execution strategies. Consequently, DT has shifted the advertising focus from creativity alone to value creation through performance measurement. Professionals now experience increasing pressure to deliver faster, measurable, and accountable outcomes within constrained resources.

#### 4.2.3 Relational tension

This tension reflects the dynamic relationship between agencies, clients and consumers. Participants described changes in agency-client relationships as clients have become more knowledgeable and actively participate in decision-making and campaign planning. However, increased involvement does not always translate into success as agencies and clients perceive digital technologies differently. As one respondent noted

“Some clients treat the digital domain as an altogether separate entity, as they fail to see it as an integrative approach where both traditional media can be integrated with digital to acquire more consumers”. (R13)

This misalignment complicates planning and execution, pressurizing professionals to justify the integrated approach.

The development of in-house agencies or newly formed specialized agencies called “new entrants or entrepreneurs” by participants increases this tension, as it challenges the presence of traditional agencies. Regarding this, respondents feel that

“If they do not adapt to new technology, somebody else will enter the market and will capture their business.” (R10)

Many national and international brands have started their own agency to eliminate intermediaries, saving both money and time. Digital communication tools have been critical to achieving this goal. This will affect the role of traditional players in the industry, pushing them to change their business models to compete. This evolving agency-client relationship puts pressure on advertising professionals to prove their relevance while adapting to changing expectations.

Furthermore, social media platforms have made communication between clients and consumers easier and more direct, reducing the agency's intermediary role. With social media teams at clients’ disposal, clients increasingly manage consumer communication and rely less on external agencies.

Although social media enhances visibility and interaction, it brings forward brand perception challenges. The respondent highlighted that

“Negative word of mouth spreads quickly on social media, affecting the client’s brand image”. (R15)

Along with this, viral content and consumer online reviews quickly change brand image among consumers. At the same time, respondents observed

“Ad fraud on social media in the form of many clickable advertisements” (R15)

which leads to declining consumer trust. “Credibility of reward” (R21) is another factor that contributes to trust issues. This forces advertising professionals to design more credible and engaging content to capture attention.

Together, these factors indicate a shift toward more complex, less stable relational structures from a dyadic agency-client interaction to a triadic relationship involving consumers. This intensifies pressure on agencies to maintain trust, relevance, and effective engagement across all stakeholders.

#### 4.2.4 Organizational tension

Finally, DT has created organizational tension through changes in work processes, professional roles, creative practices, and workplace culture within agencies. The integration of digital technologies has changed the way campaigns are conceptualized, produced, delivered, and managed. Unlike traditional formats, digital platforms impose platform-specific restrictions, enable data-driven decision-making, and support shorter-duration advertising, which changes workflows and organizational practices. In an advertising agency, being a creative organization, this tension is evident in how they change their creative processes on diverse digital platforms and formats. Traditional formats are no longer useful for digital platforms, as respondents pointed out,

“A3/A4 size brochure is now being replaced by a mobile device, which has a shorter screen size. The content written for an A3-size brochure cannot be used for mobile devices, so ultimately, the advertising copy needs to be modified.” (R5)

This change emphasizes that creativity comprises both format adaptation and idea development. Hence

“Agencies need to crack down on the code of what works and what doesn’t, which is a challenging task for agencies”. (R5)

This is critical, as social media is cluttered with brands delivering content from an Indian perspective. At the same time, consumer behavior also strengthens this tension through new creative demands. With the availability of mobile devices and various applications, consumers now prefer shorter content rather than lengthy ones, as pointed out by respondents through an example–

“Consumers prefer 15-second reels on Instagram rather than 30–60-second advertisements”. (R18)

This emphasizes that consumers' attention spans have shrunk, and agencies need to create content that grabs attention and elicits participation within this shorter timeframe. Another reason for changing consumer behavior is the abundance of content across multiple platforms, which allows them to skip any content if it doesn’t appeal to them. This implies that agencies need to deliver impactful communication. Respondent feel

“One way to do it is to give a reward big enough to lure him in”. (R19)

Such decisions depend on consumer data and performance metrics, which have reduced creative autonomy. Now creative teams need to have specialized roles such as

“Social media manager, digital strategists, PPC specialists, web analytics expert, affiliate marketing manager, SEO/SEM specialist” (R23),

to handle these new tasks, leading to fragmentation of the creative process. This pressurizes creative teams to balance creativity with speed, data insights, and platform constraints. Organizational tension is also evident through effective brand management. The respondent observed that

“Brands need to be very careful about how to manage digital media. Unawareness about digital interaction with the audience through effective appeal and well-formulated strategies can harm a brand.” (R27)

This suggests that brands may face unfavorable perception if communication via new interactive media is poor. The respondent highlighted that

“Many brands lack consistency when it comes to digital media strategies, which basically creates a barrier in brand building.” (R25)

These observations show that DT needs precise, consistent communication methods with creative innovation. Organizational work culture also belongs to this tension as digital technologies have added another layer of complexity. The shift to remote working during the COVID-19 pandemic has changed how colleagues connect, collaborate, brainstorm, and learn from each other. Participants observed that a decline in face-to-face conversation has made it difficult for knowledge sharing and exchanging creative ideas—factors that are considered important for sustaining the agency's work culture. At the same time, professionals are now expected to work on multiple tasks simultaneously with extended working hours under work-from-home (WFH) arrangements without any monetary compensation, resulting in discouragement. These changes have transformed established patterns of collaboration, leading to increasing workplace dissatisfaction.

These developments suggest that DT reshapes not only the outcomes of agencies’ work but also the processes by which agencies produce, evaluate, and manage it. Navigating organizational tension requires maintaining balance among creativity, digital media, data-driven insights, and complexities in the operations of an advertising agency.

Together, these four tensions create a multidimensional structure that reshapes capabilities, financial structures, stakeholder relationships, and working practices. These pressures compel strategic responses from the agencies, which are discussed in the following sections.

### 4.3 Adaptation mechanisms

To address professional organizational tensions, advertising agencies are reconfiguring their capabilities, processes, and structures. The findings show systematic mechanisms through which agencies adapt to the digital environment.

#### 4.3.1 Professional learning

To respond to capability tension, advertising firms are actively rebuilding their human capital. This includes not only hiring the right talent but also continuously upgrading existing employees' skill sets to meet digital needs. Participants noted that

“Hiring the right talent, talent who is receptive to change and willing to learn new media as and when required” (R33)

is important. This implies that agencies are now open to employing talent who are adaptable and eager to learn new digital technologies. Agencies want their employees to be tech-savvy and digitally skilled to remain competitive in the industry. Hence, agencies encourage their employees to participate in training programs through various channels, such as

“Workshops, online certification courses by MICA, or on platforms such as LinkedIn, organizing conferences, and interaction with external experts”. (R30)

The agency also encourages cross-functional training through interdepartmental transfers to build 360-degree knowledge. These initiatives do more than improve technical skills. They nurture creativity, strengthen planning skills, build confidence, and help professionals adapt to changing market conditions.

Advances in e-commerce platforms, OTT platforms, influencers’ social networks, AI interfaces, traditional media moving online, data privacy, and other digital disruptions have forced media professionals to engage in extensive cross-learning through team collaboration. Media professionals are now trying to hone their skills by learning as much as they can about each medium. Bringing together diverse media platforms such as print and digital helps professionals to adapt.

This forward-looking approach emphasizes anticipating upcoming technologies and being proactive rather than reactive. Overall, professional learning reveals how advertising agencies transform their human resources and technical capability.

#### 4.3.2 Technology enablement

To address the complexity of the digital environment, agencies reconfigure their work by combining digital tools, software, analytics, data-driven systems, and consumer-centric methods. Rather than relying on intuition and standardized advertising formats, agencies now depend on technology-enabled tools to support campaign planning, execution, and performance evaluation. This enablement is particularly evident in the media department, where professionals such as media planners and buyers are adapting to the digital ecosystem and trying to create synergy across all media platforms. One participant pointed out that

“Now a lot of work happens on software, which is nothing, but tech-enabled”. (R14)

Consequently, even professionals in mass media must learn software to get accurate, timely information and present it to clients to gain an advantage in planning the campaign. Such integration is further reflected in understanding consumers through continuous analysis of “real-time consumer behavior.” Another respondent noted the

“Integration of 3rd party tools to understand consumer purchase behavior” (R11)

enabling agencies to use external data systems effectively, advanced AI & ML tools, and cookie-based retargeting technology. These tools enable capabilities such as “behavioral targeting of consumers” (R34), including accurate tracking of their online presence, media choices for information consumption, needs, or pain points. This enables a shift from mass media to individual targeting, where campaigns can be tailored based on region, language, and market conditions. Another tool that helps ad agencies analyze consumer purchase behavior is transaction data from digital payments, purchase frequency, and brand performance, which helps agencies make campaigns more precise and personalized. This shift signals a move away from conventional planning, placing greater importance on consumer preferences, experiences, and buying behavior; this is why consumer journey mapping also plays an important role in choosing the right advertising platforms and crafting content strategies that resonate with consumers. Agencies can take a holistic approach by choosing appropriate digital media and the latest marketing trends for content creation. As one participant stated,

“Recognizing and creating good-quality content for the target audience is one of the ways to adapt to the new media.” (R26)

Such methods also help in evaluating campaign performance. Real-time analytics help agencies to evaluate campaign progress, assess ROI, and align operations with the client’s expectations. These changes force advertising agencies to monitor their strategies and adapt to requirements.

Technology has also strengthened organizational planning by improving integration across functional areas. Earlier separation of the creative and media departments from the mainline agency structure limited collaboration and operations, leading to fragmented consumer understanding. However, integrating shared consumer data into creative development and media planning has enabled agencies to develop a more comprehensive knowledge of the audience.

In addition, clients have shifted their focus to new communication technologies such as voice search, social media platforms, mobile apps, and new ad formats like Instagram Stories or Reels rather than plain text or images. This has affected the agency’s work, driving substantial changes in research, creative, media, production, and client management processes. One respondent explained

“The agency needs to adapt itself by looking at future trends, such as an increase in voice search. Big companies like Google & Facebook have already started using such methods. In India, within 2–3 years, infrastructure will be available, which agencies need to understand and adapt to so as not to fall behind.” (R4)

Overall, technology enablement reflects how emerging digital technologies have become embedded within advertising work at all levels. Rather than merely supporting existing practices, digital technology reshapes agencies to respond more effectively in a dynamic market environment.

#### 4.3.3 Organizational adaptation

To overcome organizational tensions, respondents feel that

“Indian advertising agencies need to break out of their boundaries and adapt to new technology and must seek a competitive advantage over their competitors”. (R5)

Advertising agencies now keep DT as a strategic priority rather than an auxiliary function, as

“More than 70% of employees are ready with and have already adapted to new technology.” (R5)

This shift is reflected in digital initiatives such as targeting customers across as many digital platforms as possible so brands do not miss consumers. Respondents feel this strategic alignment with DT should be a long-term organizational vision. In response to capability and relational tension, advertising agencies are now adopting a hybrid organizational structure that combines traditional and digital capabilities and tools. Rather than replacing traditional media entirely, agencies combine their strengths with digital media to remain competitive and balance both in response to client and market demand. Such integration of both domains has become a major trend in the advertising industry. Dual models have led to hybrid workflows to deliver campaigns across multiple channels simultaneously. However, respondents believed not all clients view hybridization as an integrative approach, indicating growing difficulty in justifying the integrated approach to clients. Another key dimension of this structural hybridization is collaboration between various digital firms. Respondents believed

“Synergy between digital specialized agencies and mainline agencies can be a great solution for adapting to digital media advertising”. (R35)

It helps to integrate expertise across different agencies. This move suggests that hybridization is not limited to internal departments but extends to external agencies, where complementary capabilities merge to manage complexity. The rise of in-house capabilities changes the operating model of advertising agencies, where agencies can act as consultants while clients handle content generation. As a result, traditional agency-client relationships have become more distributed and collaborative.

The pandemic further accelerated this shift, as WFH forced ad agencies to adopt digital technologies for daily operations. Under restricted conditions, agencies used online meetings and virtual collaboration to carry out various business functions. The agency recalibrated its approach to the new buying culture developed as businesses moved to online platforms and e-commerce models. Not just the media, but other departments of the ad agency had to adopt a new approach to managing their business. Respondents added that these collaborations remain active, as they help agencies maintain their finances. A respondent pointed out that

“Change in government policies during the pandemic, such as a ban on Chinese apps like TikTok, made in India products, etc.,” (R32)

has reinforced the need for agile operations. In such scenarios, the agency must quickly identify an alternative and make a transition to that platform. As a result, methods like “influencer marketing, especially on Instagram” (R32) became a popular choice for brands to advertise. These changes create new engagement opportunities, prompting agencies to incorporate them into their campaigns quickly. This shows agencies’ capability to reorganize work in response to changing external conditions.

Alongside these developments, professional learning initiatives also support organizational adaptation by equipping agencies with the capabilities needed to reorganize work, embrace technological change, and remain competitive, enabling firms to navigate digital ecosystems effectively.

In summary, these adaptation mechanisms enable advertising agencies to become future-oriented by integrating digital technology across advertising planning and strategies, platforms, and consumer understanding. They reflect a coordinated mechanism for countering organizational tensions. As a result, they contribute to the rise of new organizational forms and practices, which the following section explains.

### 4.4 Transformation of advertising work

The adaptation mechanisms implemented by advertising agencies collectively transform the nature of advertising work. Although these help agencies in reshaping their functioning, they do not represent a stable state or a fixed end. Rather, they reflect a provisional realignment of the agency’s strategic position in the industry.

#### 4.4.1 Professional role evolution

The increasing integration of consumer insights, data analytics, digital technologies, and new media has expanded the responsibilities of advertising practitioners beyond their traditional roles, as pointed out by a respondent

“A whole world of opportunities which are now challenging and redefining traditional roles”. (R37)

Traditional roles such as copywriter, art director, creative director, account planner, media planner and buyer have now transformed into

“Social media expert, blogger, content writer, creator, programmatic media planner, event marketer, digital media strategist, digital influencer, web designer, mobile game designer, app developer” (R39)

Rather than focusing solely on creative execution or media buying, professionals from different departments participate in campaign planning, client pitching, consumer analysis, media selection, campaign performance evaluation, client consultation, and budget allocation. Today, advertising work also needs multidisciplinary skills that combine creativity, technological know-how, analytical thinking, and better decision-making. These evolving new responsibilities redefine professional identities and elevate expectations for learning and adaptability.

#### 4.4.2 Collaborative work practices

The rise of an integrated, data-driven agency model represents an important outcome of adaptation. It brings creative, media, account, and digital functions closer while also increasing organizational complexity. Professionals now work in collaborative teams that understand consumer insights, craft compelling advertising content, and deliver campaigns that resonate closely with customers. As new media creates opportunities to produce interactive content, the need to innovate across new platforms and reach every possible audience grows, leading to artistic expression backed by meaningful data. This continuous need to coordinate multiple functions and platforms produces new capability tension for agencies. While agencies must adopt these advanced tools, they must also maintain cross-functional expertise. This doesn’t eliminate pressure; it renews the challenge and requires professionals to be adaptable.

#### 4.4.3 Adaptive work culture

Operational agility enhances the agency’s responsiveness and adaptability to market changes more efficiently. However, this increased agility pushes professionals to work in an environment of new pressures, including constant monitoring, rapid decision-making, continuous adjustment, real-time data systems, flexible workflows, and digital tools. This intensifies workload demands, increases reliance on digital technology, and increases collaboration with digital entrants. Instead of temporary adjustments, these practices are more rooted in everyday organizational life, creating both capability and organizational tensions.

## 5 Discussion

### 5.1 Synthesis of findings

Based on qualitative interviews with advertising professionals in the Indian Advertising Industry, this article illustrates how they comprehend and navigate DT in the advertising industry. Our findings show that DT is fundamentally reshaping advertising through ongoing interactions among four core themes.

In line with prior research, the study identifies technological advancement, varying client expectations, and changes in the media landscape as key drivers of transformation in the advertising industry (Hughes and Vefeas, 2019; Patwardhan et al., 2019; Stuhlfaut and Windels, 2019; Mokoena et al., 2023). However, they do not operate individually; instead, they interact and continuously challenge existing work practices and organizational routines. This creates new professional and organizational tensions that are not just obstacles to technological adoption, but an inherent consequence of operating in a digital organizational environment. This extends to previous studies focused on internal challenges linked with DT (Childers et al., 2018; Patwardhan et al., 2019; Kumar Sharma et al., 2020; Iyer and Bright, 2024).

Notably, the findings show that industry’s responses to these new tensions are part of an interconnected mechanism that enables agencies to stay relevant, build digital competences, and tackle new tensions, supporting existing literature (Figueiredo et al., 2021; Iyer and Bright, 2024; Ceccotti et al., 2025). Therefore, adaptation appears as a constant organizational process rather than a finite transformation. These adaptive responses reveal broader outcomes of DT, highlighting prior research on how advertising work is expanding by Hughes and Vefeas (2019) and Mokoena et al. (2023). In this digital era, professionals must blend their creative, technological, analytical, and digital capabilities for everyday tasks, creating new competency and organizational demands. Therefore, transforming advertising work creates conditions for further adaptation. This reflects recursive and continuous process.

### 5.2 Theoretical implications

The current study also adds to the theoretical understanding of how digital technologies reveal workplace transformation in different ways. First, the results support Sociotechnical Theory (Ghosh et al., 2025) by showing that DT emerged through continual interplay between technological innovations and changes in organizational, professional, and social dynamics rather than a one-time change. Digital tools and systems reshape what professionals do, how they plan and get work done, and how organizations are structured, highlighting the interdependence of social and technical structures. Second, the study also adds to Organizational paradox theory (Klein et al., 2024) by showing that capability gaps, performance pressure, relationship dynamics, and organizational tensions are not temporary obstacles but permanent aspects of DT. Rather than being fully solved, they create new tensions among creativity, efficiency, autonomy, and technological reliance, which employees and organizations must continue to balance. Third, the findings resonate with Adaptive structuration theory (Hsiao, 2026) by highlighting that organizations do not simply adapt to technology; they weave these technologies into daily routine tasks, creating new structures and changing professional roles. Based on these views, the study conceptualizes a Recursive Tension—Adaptation Framework. Unlike linear models, this framework views organizations as dynamic systems shaped by nonlinear interplay among the four themes and continual adaptation. In this way, the framework offers a process-oriented justification of how organizations in the service industry navigate DT continuously through obstacles and opportunities.

### 5.3 Practical implications

Our findings have important real-world implications for industry stakeholders, such as agency leaders and managers steering DT. Managers need to recognize that DT is not just a technological initiative but a comprehensive organizational change that reshapes an industry. The agency must invest in cutting-edge digital tools, employee development, workflow revamps, and organizational support systems. The agency should foster a work culture of continual learning, cross-functional collaboration, and open communication to help employees adapt. Regularly monitoring and updating organizational practices will help agencies stay agile and responsive to changing structures, skills, and work processes, making them sustainable in a competitive market. The research also emphasizes the need for media and business schools to update their curricula to align with the latest market trends. Stronger industry-academia partnerships can prepare well-equipped digital talent.

### 5.4 Limitations and future scope

Despite its contribution, the study has its limitations. A qualitative inquiry was conducted from an Indian perspective based on professional’s self-reported experiences, which may limit the applicability of the results to other industries and countries with varying levels of digital maturity. The study does not include views from other stakeholders, such as clients, technology providers, and academic institutions, which provides avenues for future research. Researchers may investigate comparative studies across countries to gain a broader perspective. Future research may employ longitudinal approaches to track the evolution of DT and may test the proposed recursive tension adaptation framework.

## 6 Conclusion

The current study investigated how Indian advertising practitioners comprehend and navigate the changing digital landscape. The results suggest a dynamic, ongoing process rather than a straightforward linear shift. Agencies continually reorganize their working strategies, structures, skills, and daily operations to keep pace with technological change. Each step of adaptation introduces new challenges for professionals, inducing continuous change. This suggests that digital transformation is a continual renewal process. Practically, for agencies, this means prioritizing flexibility, making data-informed decisions, and encouraging adaptability to remain competitive. Overall, the study provides an advertising professional-centric and process-oriented perspective that adds to the comprehension of digital transformation in the Indian context.

## Data Availability

The datasets presented in this study can be found in online repositories. The names of the repository/repositories and accession number(s) can be found below: https://doi.org/10.6084/m9.figshare.31988499.v5.
