# Supplementary material for: Understanding adaptation to digital transformation: evidence from Indian advertising industry
Source: Front Sociol. 2026 Oct 28;11:1953897. doi: 10.3389/fsoc.2026.1953897 (PMC13751470; doi:10.3389/fsoc.2026.1953897)
Supplement: Supplementary file 1 [file Table_1.DOCX]

Supplementary Material

Table 4. Illustrative example of theme development using inductive TA.

| **Theme – Adaptation Mechanism** | | | |
| --- | --- | --- | --- |
| **Quotations** | **Initial codes** | **Categorized codes** | **Subthemes** |
| You have to be updated each and every day so. So whenever platforms changes you need to learn any new functionality they have added. We need to spend time to understand how it works and implement it. Every time Google comes with different algorithm, we need to modify our website and make changes for every client. It is just trial and error things. | Learning,  Adaptation | Job, Learning, Skill | Professional Learning |
| So my current role is I'm a group head and I do client strategy and servicing. So servicing is just dealing with the client on day-to-day basis getting their requirements. Client strategy is a bigger umbrella wherein we work on campaigns to gain more followers to generate ROI | Job Responsibilities |  |  |
| He should be creative. Always thinking of solutions. Hungry to learn and eager to try. Experience does ads maturity but creativity can come from anywhere | Learning,  Open Mindedness |  |  |
| not most of the colleges do this, but they started doing it, having guest lecturers on board and so they can actually tell them examples wherein they faced an issue and how they've overcome it | Education,  Industry Expert |  |  |
| See in terms of challenges, there are multiple things that runs, but I think the biggest challenge that we as agencies find is getting the right talent in the market. | Talent | Recruitment |  |
| So if tomorrow I'm interviewing an executive or an intern, I would want them to be very, very active on say, a Snapchat. Or a clubhouse. So these might be 2 platforms I am not very active on or I do not really like it because I'm not that generation who has grown up with Snapchat. I might be the Instagram and Facebook generation. So with digital, if you are in this space and you're working over here, it is an ever evolving one and you need to be up to date with whatever new is coming up. So the moment you are not up to date you will hit rock bottom, so it's like a learning every day even though I'm not that snapshot generation, I would still go back to my cousins or or someone who I have in the family who is 20 years. old or the exact and be like OK, how do you do this? Teach me. So it is that space where you need to keep learning and relearning and there is no stopping when it comes to learning on digital. | Work Adaptation,  Professional Development  Learning,  Skill Acquisition,  Digital skills,  Digital Media | Adaptation |  |
| Now it is more like at the back of our heads that these are the platforms and this is what they offer. If there is something new that comes in, we try and give it a, like Twitter had stories, Instagram had it too. Then when Instagram got taken over by Facebook and it came onto Facebook, Snapchat and Stuff like that. | Adaptability |  | Technology Enablement |
| Everything is done using computers, sometimes even mobile phones. User generated content is mostly on mobile; and shared freely on social media. The line between creator and consumer has disappeared. It is two-way communication which has taken over, sometimes even three way, between a brand, its consumers and the agency. Agencies are also under threat, because the client can produce their own communications (especially on social media), and also ropes in consumers for content. | Social Media  Consumer Behavior | Technology |  |
| A shift in focus to behavioural targeting. Digital has enabled accurate tracking of people’s media and online behaviour, as well as what they buy (transaction data) and where they buy (location / retail data). This allows advertisers to target individuals, based on their search, sharing and purchase behaviour – as opposed to the mass media approach earlier. | Data Analytics  Consumer Behavior |  |  |
| So it's always advisable, or it is actually very good practice to go ahead with single agency and that's the reason why a lot of agencies are now trying to integrate the digital phenomena as well, because you know, they realize that digital advertising is essential for growth of a business. Agencies have to adapt and if they don't adapt, they will die down. It's quite simple as that. | Business Model | Organization | Organizational Adaptation |
| Actually, no, because the agencies who were doing offline earlier they have recently started their online wing. | Hybrid Model |  |  |
| But now when 2020 hits offline agencies weren't prepared for such kind of a hit. That is when most of them started developing their digital wing. | Digital Transformation  Agency Structure |  |  |
| My agency consists of two wings. Mainline and Digital. The mainline wing designs the strategy and campaign and the digital wing has to work with us in creating a digital leg to the same campaign | Organizational Structure  Collaboration |  |  |

Initial codes were derived from participant’s quotations. Similar codes were grouped together to form categorized codes based on which main theme was defined. The table shows the development of theme – Adaptation Mechanism
